# Association between day of delivery and obstetric outcomes: observational study

**DOI:** 10.1136/bmj.h5774

**Published:** 2015-11-24

**Authors:** William L Palmer, A Bottle, P Aylin

**Affiliations:** 1 Dr Foster Unit, Department of Primary Care and Public Health, Imperial College London, London SW7 2AZ, UK; 2 National Audit Office, London, UK

## Abstract

**Study question** What is the association between day of delivery and measures of quality and safety of maternity services, particularly comparing weekend with weekday performance?

**Methods** This observational study examined outcomes for maternal and neonatal records (1 332 835 deliveries and 1 349 599 births between 1 April 2010 and 31 March 2012) within the nationwide administrative dataset for English National Health Service hospitals by day of the week. Groups were defined by day of admission (for maternal indicators) or delivery (for neonatal indicators) rather than by day of complication. Logistic regression was used to adjust for case mix factors including gestational age, birth weight, and maternal age. Staffing factors were also investigated using multilevel models to evaluate the association between outcomes and level of consultant presence. The primary outcomes were perinatal mortality and—for both neonate and mother—infections, emergency readmissions, and injuries.

**Study answer and limitations** Performance across four of the seven measures was significantly worse for women admitted, and babies born, at weekends. In particular, the perinatal mortality rate was 7.3 per 1000 babies delivered at weekends, 0.9 per 1000 higher than for weekdays (adjusted odds ratio 1.07, 95% confidence interval 1.02 to 1.13). No consistent association between outcomes and staffing was identified, although trusts that complied with recommended levels of consultant presence had a perineal tear rate of 3.0% compared with 3.3% for non-compliant services (adjusted odds ratio 1.21, 1.00 to 1.45). Limitations of the analysis include the method of categorising performance temporally, which was mitigated by using a midweek reference day (Tuesday). Further research is needed to investigate possible bias from unmeasured confounders and explore the nature of the causal relationship.

**What this study adds** This study provides an evaluation of the “weekend effect” in obstetric care, covering a range of outcomes. The results would suggest approximately 770 perinatal deaths and 470 maternal infections per year above what might be expected if performance was consistent across women admitted, and babies born, on different days of the week.

**Funding, competing interests, data sharing** The research was partially funded by Dr Foster Intelligence and the National Institute for Health Research (NIHR) Imperial Patient Safety Translational Research Centre in partnership with the Health Protection Research Unit (HPRU) in Healthcare Associated Infection and Antimicrobial Resistance at Imperial College London. WLP was supported by the National Audit Office.

## Introduction

Previous studies, across a range of countries, have identified higher mortality in patients admitted on weekends (compared with weekdays) across a range of medical conditions—a phenomenon termed the “weekend effect.”^1^ ^2^ ^3^ ^4^ ^5^ ^6^ This calls into question the idea that quality of care is equal irrespective of when someone presents at hospital. However, not all studies have identified an association between poor outcomes and out of hours periods.^7^ ^8^ ^9^

MacFarlane published a paper in 1978 that showed a seven day cycle in birth numbers across England (and Wales) and that perinatal mortality was higher among babies born at weekends.^10^ Similar studies in the 1970s found similar phenomena in other developed countries.^11^ ^12^ The delivery of obstetric care has changed dramatically since that time; however, where the weekend effect has been evaluated, this has predominantly been based on mortality. In setting out key challenges in obstetric care—albeit in a broader, global context—a paper from the World Health Organization highlighted ineffective referral to, and inadequate availability of, 24 hour quality services to emergency obstetric care services.^13^

We investigated the association between day of delivery and the quality and safety of care and, in particular, compared weekend with weekday performance. We also explored the association between outcomes and staffing levels.

## Methods

### Data sources

We extracted the details of deliveries in English NHS public services from 1 April 2010 to 31 March 2012 from the Hospital Episode Statistics database. The database consists of individual entries each covering the continuous period during which the patient is under the care of one consultant. We linked these episodes of care together to create a single record for each admission, including cases in which a mother or baby was transferred to another NHS service. Diagnoses are recorded using ICD-10 (international classification of diseases, 10th revision), and procedures are coded using OPCS-4 (Office of Population Censuses and Survey’s classification of surgical operations and procedures, fourth version).

We also extracted information recorded on potential confounders: age of the mother, baby’s sex, parity (maternal indicators only), multiple delivery, socioeconomic deprivation (fifth of Carstairs deprivation score),^14^ previous caesarean section (maternal only), ethnic group, gestational age, birth weight, delivery method, and other maternal conditions (pre-existing diabetes, gestational diabetes, pre-existing hypertension, pre-eclampsia or eclampsia, placenta praevia or abruption, polyhydramnios, oligohydramios). When characteristics were not recorded comprehensively, we treated missing data—either recorded as “unknown” or not ascertained—as a separate value and included them in the analysis (except in some sensitivity models, as described below). To investigate the risk of residual confounding bias, we did some sensitivity analyses on our results by using additional case mix variables (such as induction of labour), different exclusion criteria (for example, removing non-cephalic deliveries), alternative regression models (for example, hierarchical), only cases with comprehensive known case mix variables (that is, no missing values), and variants on the outcome measures (for example, evaluating immediate neonatal deaths). Around 37 000 maternities were to the same women (that is, they gave birth twice during the study period); as this was a small proportion (2.8%) and the adverse events were rare, we could not create a robust hierarchical modelling to account for this clustering. However, when results were significant, we removed these cases and reanalysed performance as an additional sensitivity check.

Data on hours of consultant presence on the labour ward came from two previous audits, by the National Audit Office and the Royal College of Obstetricians and Gynaecologists.^15^ ^16^ We linked data by using unit names with 100% completeness. We created a binary indicator of compliance with recommendations made by the Royal College, which was based on a recommended minimum consultant presence per week of 60 hours for units with 2500 to 4000 births, 98 hours for 4000 to 5000 births, and 168 hours for more than 5000 births. We restricted the analysis to obstetric units and sites for which data were reported for the obstetric and midwifery led unit combined.

### Statistical analysis

The indicators used for this study covered both maternal and neonatal outcomes and are defined in table 1. We chose the indicators and confounding variables used in the case mix model on the basis of a comprehensive review of the literature and also data availability.

**Table 1 tbl1:** Details of quality and safety indicators

Indicator	Rationale	Definition*	Exclusions
**Maternal**			
Perineal tear	Third and fourth degree tears during vaginal delivery are not all preventable, but risk can be reduced through appropriate labour management and care standards^17^	Admission includes both diagnosis (ICD-10: O702-3) and procedure code (OPCS: R322, R325)	Caesarean sections
Puerperal infection	Puerperal sepsis can be associated with poor care (especially hygiene) at time of birth^18^	Admission includes diagnosis code for puerperal sepsis (ICD-10: O85-6) within 42 days of birth	None
Three day emergency readmission (all cause)†	Readmission rates have previously been shown to be associated with hospital care provided^19^	Emergency inpatient admission within 3 days, for which “admission source” field does not suggest transfer from other provider	Births for which discharge date is in final month of year covered in data year (March)
**Neonatal**			
In-hospital perinatal mortality	Rate of still births plus in-hospital deaths within 7 days is associated with how hospital care is provided^20^	Value suggesting stillborn in birth status (2, 3, 4) or discharge fields (5), and discharged within 7 days with discharge method (4) suggesting death	None
Injury to neonate	Although rare, birth trauma to neonate is often preventable^21^	Injury diagnosis code within admission (ICD-10: P102-4, P108-12, P114-5, P119, P122, P130-1, P138-9, P142, P148-9, P15)	Premature births, as identified through diagnosis code (ICD-10: P070-3), gestational age (<28 weeks), or birth weight (<2.5 kg)
			Injury of skeleton (osteogenesis imperfecta diagnosis, ICD-10: Q780)
			Stillborn babies
Selected neonatal infections	Neonatal infection is associated with poor care (especially hygiene) at time of birth^18^	Diagnosis procedure code (ICD-10: P36, P372/5/8-9, O753, O85-6, A41, A32, A49) within 3 days	Premature births, as identified through diagnosis code (ICD-10: P070-3), gestational age (<28 weeks), or birth weight (<2.5 kg)
			Stillborn babies
Three day emergency readmissions (all cause)†	Readmission rates have previously been shown to be associated with how hospital care is provided^22^	Emergency inpatient admission within 3 days, for which “admission source” field does not suggest transfer from other provider	Births for which discharge date is in final month of year covered in data year (March)
			Missing birth discharge date (~1% of births)
			Death during birth admission

*Discharges meeting inclusion/exclusion criteria for denominator. Records extracted according to the following case ascertainment rules. Delivery episodes: valid delivery method in either procedure (OPCS R17-R25 in any of the operation/procedure fields) or delivery method recorded in the maternity tail (delmeth=0-9); birth episodes: episode type recorded as 3 or 6 or admission methods recorded as 82 or 83.

†Literature review identified longer time periods for readmission rates; however, for purpose of this day of the week study, three day rates were used in keeping with suggestion that it is a more appropriate timeframe to evaluate association between day of admission and mortality.^6 31^

We applied the indicator definitions to the data extract to obtain denominator and numerators, categorised by day of admission (for maternal indicators) or birth (for neonatal records) rather than by day of complication; for simplicity, we use “day of delivery” in this paper. We calculated national performance across the measures, disaggregated by day of delivery. We also analysed results by weekend versus weekday performance, defining the weekend as the period from midnight on Friday to midnight on Sunday and weekdays as all other times. We used multiple logistic regression to adjust for the effects of covariates. We did not adjust for the clustering of patients within hospitals (or community maternity services) in the model results described in the paper, as the hospital level effects were found to be small (and did not materially affect the key findings reported here), except when we included site level data (hours of consultant presence) as described explicitly below.

We also show results by plotting adjusted odds ratios and 95% confidence intervals by day of delivery, using Tuesday as an a priori reference. As described in the discussion, we chose this reference day for consistency and to reduce any effect on the measured performance of the reference day from longer admissions in which care was received at the weekend.

For in-hospital perinatal death and puerperal infections (maternal), we repeated these regression techniques for just cases on the reference days (Tuesdays). By applying the resulting (Tuesday specific) estimated effects of mother’s or neonate’s characteristics to the other (Wednesday to Monday) cases, we calculated estimates for the outcomes as if those non-Tuesday cases had had similar rates to their Tuesday counterparts.

We used SAS version 9.2 for analyses, using the PROC LOGISTIC and PROC GLIMMIX procedure for regression analyses.

### Patient involvement

The study design was developed following a literature review. No patients were involved in setting the research question or the outcome measures; nor were they involved in the design and implementation of the study. There are no plans to involve patients in the dissemination of results.

## Results

Between April 2010 and March 2012, we identified 1 332 835 maternities and 1 349 599 births. The most common adverse event was perineal tear (3.0%), and the least common was maternal readmissions (0.2%) (table 2).

**Table 2 tbl2:** Number of births and maternities and complication rates

Measure	No of events (deliveries) included	Proportion of cases* (%)
Maternities:		
Perineal tear	30 548 (1 004 515)	3.0
Puerperal infection	11 128 (1 332 835)	0.8
Three day maternal emergency readmissions	2471 (1 269 265)	0.2
Births:		
In-hospital perinatal mortality	8999 (1 349 599)	0.7
Injury to neonate	18 316 (1 245 419)	1.5
Selected neonatal infections	26 752 (1 343 593)	2.0
Three day neonatal emergency readmissions	15 299 (1 281 557)	1.2

*Expressed as percentage of cases meeting inclusion criteria.

The distribution of births was not even across the days of the week. The most common day for giving birth was Thursday (15%; 206 732 births and 205 632 maternities), and the least common was Sunday (12%; 167 159 births and 159 132 maternities). On average, 21% fewer maternities took place each day at weekends (160 400) compared with weekdays (202 400); however, if elective (planned) caesarean sections are excluded the difference falls to 11% (157 900 *v* 176 800). This variation in delivery methods is one of the most material differences in the characteristics of the study population, as set out in table 3. Although some case mix factors such as delivery method were recorded comprehensively, other characteristics were susceptible to missing data (either recorded as “unknown” or not ascertained), most notably 8% of maternal ethnicity, 13% of gestational age, and 10% of birth weights.

**Table 3 tbl3:** Characteristics of study population*. Values are percentages (numbers)

Characteristic	Weekdays (n=1 012 119)	Weekends (n=320 716)	P value
Delivery method:			<0.001
Spontaneous vertex	59.7 (603 770)	68.6 (219 858)	
Spontaneous, other cephalic	0.5 (4700)	0.5 (1719)	
Low forceps, non-breech	3.9 (39 856)	4.6 (14 771)	
Other forceps, non-breech	2.1 (21 320)	2.5 (7858)	
Ventouse, vacuum extraction	6.1 (61 351)	6.9 (22 097)	
Breech	0.4 (4268)	0.4 (1417)	
Breech extraction, not otherwise specified	0.1 (546)	0.1 (158)	
Elective caesarean	12.7 (128 059)	1.5 (4845)	
Emergency caesarean	14.6 (147 627)	14.9 (47 788)	
Other	0.1 (622)	0.1 (205)	
Maternal age (years):			<0.001
<19	5.1 (51 850)	5.8 (18 609)	
20-24	18.4 (186 359)	19.9 (63 889)	
25-29	27.5 (278 540)	28.2 (90 557)	
30-34	28.6 (289 937)	28.2 (90 374)	
35-39	16.2 (164 047)	14.6 (46 728)	
≥40	4.1 (41 386)	3.3 (10 559)	
Ethnicity:			<0.001
White	72.3 (731 968)	71.8 (230 123)	
Asian	10.3 (104 552)	10.5 (33 565)	
Black (including black British, Afro-Caribbean)	5.1 (51 678)	5.0 (15 960)	
Mixed	1.5 (15 033)	1.5 (4827)	
Other (including Chinese)	3.3 (33 073)	3.4 (10 742)	
Unknown/not stated	7.5 (75 815)	8.0 (25 499)	
Carstairs deprivation fifth:			
1 (least deprived)	15.2 (154 180)	14.9 (47 870)	<0.001
2	16.3 (164 915)	16.0 (51 404)	
3	19.0 (192 353)	18.9 (60 735)	
4	22.0 (223 148)	22.3 (71 498)	
5 (most deprived)	26.5 (268 635)	27.0 (86 611)	
Unknown	0.9 (8888)	0.8 (2598)	
Primiparous	43.5 (439 994)	46.4 (148 751)	<0.001
Gestational age (weeks):			<0.001
<37	7.6 (76 578)	7.4 (23 628)	
37-39	35.7 (361 190)	30.3 (97 062)	
40-41	39.7 (402 103)	45.1 (144 803)	
≥42	3.6 (36 144)	4.1 (13 061)	
Unknown	13.5 (136 104)	13.2 (42 162)	
Birth weight (g)			<0.001
<2500	5.9 (60 114)	5.4 (17 240)	
2500-4000	74.1 (749 757)	74.7 (239 644)	
≥4000	10.0 (101 694)	10.2 (32 580)	
Unknown	9.9 (100 554)	9.7 (31 252)	
Multifetal gestation:			<0.001
Yes	1.6 (16 263)	0.9 (2887)	
No	91.0 (921 329)	91.8 (294 400)	
Unknown	7.4 (74 527)	7.3 (23 429)	
Previous caesarean section†	12.8 (129 427)	6.8 (21 955)	<0.001
Induction of labour rates	32.1 (324 627)	32.6 (104 416)	<0.001

*Data are for maternities and are categorised by day of admission.

†Calculated using data extracted on caesarean sections since April 1996.

Table 4 shows the results of the association between day of delivery and performance in the seven measures of quality and safety when we compared weekday and weekend rates. We found statistically significant associations in four of the indicators, all of which were consistent with a lower standard of care for women admitted and babies born at weekends. The largest effects were seen in the higher rates of perinatal mortality (adjusted odds ratio 1.07, 95% confidence interval 1.02 to 1.13), puerperal infections (1.06, 1.01 to 1.11), and injury to neonate (1.06, 1.02 to 1.09).

**Table 4 tbl4:** Comparison between weekday and weekend deliveries (babies) or admissions (women) across indicators of quality and safety of care

Indicator	Weekday			Weekend		P value	Odds ratio (weekday as reference)	
	Unadjusted % (No)	Adjusted rate (%)		Unadjusted % (No)	Adjusted rate (%)		Unadjusted	Adjusted (95% CI)
Perineal tear	3.03 (22 299/736 433)	3.04		3.08 (8249/268 082)	3.05	0.812	1.01	1.00 (0.98 to 1.03)
Puerperal infection	0.83 (8358/1 012 119)	0.82		0.86 (2770/320 715)	0.87	0.010	1.03	1.06 (1.01 to 1.11)
Three day maternal readmissions	0.20 (1926/963 094)	0.20		0.18 (545/306 171)	0.18	0.135	0.90	0.93 (0.84 to 1.02)
In-hospital perinatal mortality	0.64 (6481/1 006 765)	0.65		0.73 (2518/342 834)	0.71	0.007	1.14	1.07 (1.02 to 1.13)
Injury to neonate	1.43 (13 278/928 529)	1.45		1.59 (5038/316 890)	1.53	0.002	1.11	1.06 (1.02 to 1.09)
Selected neonatal infections	1.99 (19 900/1 002 476)	1.99		2.01 (6852/341 117)	2.00	0.542	1.01	1.01 (0.98 to 1.04)
Three day neonatal readmissions	1.19 (11 323/955 357)	1.18		1.22 (3976/326 200)	1.23	0.044	1.02	1.04 (1.00 to 1.08)

Over the seven indicators, we found 10 examples of statistically significant differences in performance across days in comparison with Tuesday. Three of the indicators had no statistically significant variations in performance, whereas perinatal mortality had five days (all except Monday) with worse performance than the reference day (figure[Fig fig1]).

**Figure fig1:**
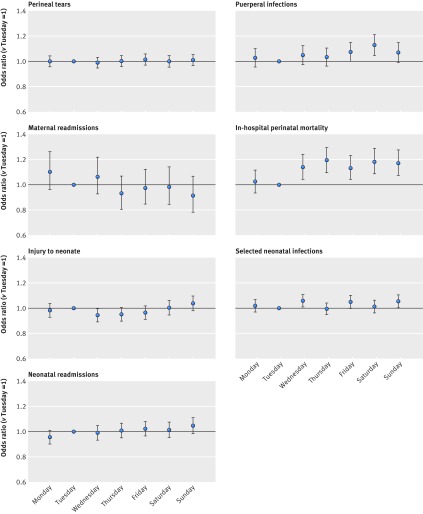
Association between performance and day of women’s admission or babies’ delivery.

Within the maternity data extract, 51 (39.8%) of the 128 units were compliant with the recommended level of consultant presence. We found statistically significant differences in rates of perineal tears between compliant (2.95%) and non-compliant (3.29%) units (table 5). We found no statistically significant differences across the other measures.

**Table 5 tbl5:** Association between compliance with consultant staffing levels and indicators of quality and safety of care

Measure	Unadjusted % (No)			Adjusted	
	Compliant	Non-compliant		Odds ratio (95% CI)*	P value
Perineal tear	2.95 (3916/132 742)	3.29 (9443/286 868)		1.21 (1.00 to 1.45)	0.048
Puerperal infection	0.76 (1345/176 913)	1.07 (4149/386 746)		0.91 (0.54 to 1.56)	0.737
Three day maternal readmissions	0.21 (338/161 699)	0.17 (614/352 829)		1.03 (0.69 to 1.53)	0.892
In-hospital perinatal mortality	0.60 (1026/170 843)	0.75 (3076/410 957)		1.21 (0.85 to 1.72)	0.289
Injury to neonate	1.59 (2513/158 443)	1.46 (5481/376 164)		0.83 (0.46 to 1.49)	0.533
Selected neonatal infections	2.04 (3468/170 169)	2.28 (9342/408 886)		1.31 (0.79 to 2.18)	0.296
Three day neonatal readmissions	1.25 (1935/154 769)	1.19 (4405/371 462)		1.03 (0.78 to 1.37)	0.834

*Odds ratio of performance of non-compliant versus compliant unit (as reference).

We estimated that 770 (95% confidence interval 720 to 830) more perinatal deaths per year, from the annual total of 4500 deaths among 675 000 births, occurred above what we would expect if mortality was always the same as for babies delivered on a Tuesday. We also found 470 (430 to 510) maternal infections, from the annual total of 5569 events across 666 400 maternities, above what would be expected from performance seen for women admitted on the reference day.

## Discussion

This study highlights an association between day of delivery and aspects of performance; in particular, babies born at the weekend had an increased risk of being stillborn or dying in hospital within the first seven days. Moreover, the results also suggest increases in the rates of other complications for both women admitted and babies born at weekends, with higher rates of puerperal infection, injury to neonate, and three day neonatal emergency readmissions.

### Strengths and limitations of study

The study has several strengths. Firstly, it represents the most comprehensive assessment of its type of the “weekend effect” in obstetric care, by evaluating performance against a range of outcome measures. The approach taken also benefits from using the Hospital Episode Statistics database, which has the advantage of being longitudinal and timely, covering all hospital admissions, and being relatively cheap, costing £1 (€1.39; $1.53) per record to collect compared with around £10-£60 per record for clinical registers.^23^ Such data are, however, susceptible to shortcomings in ascertainment and reporting of complications. Specifically, missing data and inaccurate coding (especially in diagnostic fields) can be a problem—for example, gestational age was missing from 13% of cases—although, as a validation check, we reanalysed key results by removing cases with missing data. However, the coding process is not influenced by day of delivery, mitigating the risk of measurement bias in the comparisons made. In addition, a recent systematic review has shown data accuracy to be improving.^24^

A key limitation of the analysis is our method for categorising performance temporally. The data used in this study did not include information on the time of admission, so we were not able to investigate the wider question of the quality of out of hours care in this study. The neonatal indicators use date of birth to assign the day even though the outcome might be affected by the standard of care given to the mother during labour, with 29% of births occurring the day after maternal admission.^25^ Thus, Monday’s performance might be substantially affected by quality of care over the weekend for non-same day births. Similarly, for maternal indicators, which are categorised by day of admission, Friday’s performance might also be substantially affected by weekend care. To mitigate this, we made a decision to use a midweek reference day, choosing Tuesday a priori, and for consistency we used this same day for both neonatal and maternal indicators. This problem of how births are categorised temporally is relevant to perinatal mortality as an indicator, as it may be influenced by the care received up to seven days after birth. This may explain why mortality was raised for deliveries on Wednesday to Friday as well as at the weekend. To investigate this further, we also analysed differences in the rate of immediate neonatal death (on the day of birth), which also showed significantly raised mortality at weekends (adjusted odds ratio 1.09, 95% confidence interval 1.03 to 1.15). Future work might focus on the influence on outcomes of out of hours care on the days subsequent to delivery.

A further limitation relates to the well recognised influence of maternal and fetal risk factors on outcomes.^26^ As set out in the results, even after exclusion of elective caesarean sections, an average of 11% fewer maternities took place at weekends compared with weekdays. Evidence of a weekend cycle is not new, but it suggests possible differences in case mix between days, which were also highlighted in the comparison of the study population’s characteristics (table 3). We accounted for case mix by using mother level or neonate level logistic regression to calculate the expected number of events for outcome measures. To investigate the risk of residual confounding bias, we did some sensitivity analyses as described in the methods section. None of these reanalyses had a material effect on the significance of the key findings. However, the administrative database used gives only limited information on the complexity of the delivery, and some important case mix factors, such as maternal obesity and smoking,^26^ are not recorded. The literature is inconclusive on the extent to which this limitation might affect the results.^27^ ^28^

Not all of the complications are avoidable. For example, within the perinatal deaths identified in the study, antepartum stillbirths may be less preventable than others. However, previous findings on the extent of variation in performance between providers suggest that the indicators are amenable to the care provided.^15^ ^16^ Reasons also exist to suggest that the inequality of care is more pronounced than suggested by this study. If the effect is caused by a staff deficiency and a lack of resources, one would expect poorer quality and safety at all out of hours periods during the week, including bank holidays and weekday evenings and nights. If this is the case, the out of hours periods during weekdays are masking some the effect. There are several possible explanations for these findings, including a lack of consultant obstetrician presence. This study has provided some evidence to support the theory that one of the contributing factors to the weekend effect might be a failure to meet recommended levels of consultant presence, with a significant association between staffing and perineal tear rates. This association suggests that the weekend effect might be amenable to the provision of healthcare. Confidence intervals for relations with the other outcomes were very wide, suggesting no clear association. No data were available to allow us to investigate any effect of differing midwifery staffing levels by day of the week.

### Comparison with other studies

Gould and colleagues analysed administrative data from 1.6 million live births between 1995 and 1997 in the United States and found raised levels of neonatal mortality at weekends. Specifically, they observed a neonatal mortality rate of 2.8 per 1000 weekday births compared with 3.1 for weekend births (odds ratio 1.12, 1.05 to 1.19). However, after adjustment for birth weight, the differences were no longer significant.^29^ This rejection of the hypothesis of greater complications at weekends has been reiterated elsewhere, including by research in Canada and the United States.^27^ ^30^ However, other studies have identified evidence of a weekend effect.^11^ ^27^ In particular, a study in Scotland found an adjusted odds ratio for weekend neonatal death of 1.3 (1.0 to 1.6), compared with weekday in-hours, which was similar for all out of hours deliveries 1.3 (1.1 to 1.6).^28^ Given the differences in structures, resourcing, and management of health services internationally, this mixed picture is expected. These studies, however, have primarily focused on a limited range of outcomes and therefore could not capture some of wider aspects of the quality and safety of care evaluated in this research.

### Conclusions and policy implications

Further work is needed to understand what organisational factors might influence the weekend effect and to investigate centres that have reduced the disparities in access and outcome in out of hours care. A starting point for this would be to allow services to compare how they resource out of hours maternity services and, where data permits, the extent of the weekend effect in their organisation with that of their peers. Additional analysis might also involve the use of large clinical audit databases in which time of delivery might be better recorded. Other future work to add to this analysis could include further sensitivity analysis by removing difficult cases and therefore reducing potential bias in case mix. For instance, the analysis could be repeated excluding cases with gestational age outside 37-43 weeks, perinatal deaths ascribed to congenital abnormality or rhesus isoimmunisation, and stillbirths.^28^ Other techniques such as propensity score matching could also be considered. The time of onset of the complication as well as the time of delivery could also be analysed. For instance, previous work has looked at the date of death as well as the date of birth,^12^ but interpreting the date of death from antepartum and intrapartum stillbirths is difficult.^27^ Another possibility would be to look at all out of hours periods; the existing literature is inconsistent on how to account for holiday periods.^10^ ^12^ ^29^

Scope also exists to extend this analysis to other specialties; similar results have been found in a limited number of other clinical areas, such as stroke, pulmonary embolism, hip fractures, and upper gastrointestinal haemorrhage.^6^ ^31^ A greater understanding of the problem will also require better data, and the inclusion of an out of hours admission flag for hospital administrative data should be considered. Unless managers and practitioners work to better understand and tackle the problems raised in this paper, health outcomes for mothers and babies are likely to continue to be influenced by the day of delivery.

**What is already known on this topic**

Studies on whether obstetric outcomes are associated with day of delivery have given conflicting results

**What this study adds**

This paper provides an evaluation of the “weekend effect” in obstetric care, covering a range of outcomes
The possible scale of the problem is highlighted, for example, in the highly statistically significant increase in perinatal mortality at the weekend
The results would suggest approximately 770 perinatal deaths and 470 maternal infections per year above what might be expected if performance was consistent across women admitted, and babies born, on different days of the week

## Notes

Contributors: All authors were involved in the study concept and design and in the acquisition, analysis, and interpretation of data. WLP and AB did the statistical analysis. PA supervised the study and provided administrative, technical, and material support. WLP drafted the manuscript, and all authors critically revised it for important intellectual content. All authors were involved in amending and finalising the manuscript and have approved the final version of the paper. WLP is the guarantor.

Ethical approval: The Confidentiality Advisory Group granted permission under Section 251 of the NHS Act 2006 (formerly Section 60 approval from the Patient Information Advisory Group) to hold confidential data and analyse them for research purposes (PIAG 2-05(d)/2007). The South East Ethics Research Committee gave approval to use them for research and measuring quality of delivery of healthcare (10/H1102/25).

Transparency declaration: The lead author (the manuscript’s guarantor) affirms that the manuscript is an honest, accurate, and transparent account of the study being reported; that no important aspects of the study have been omitted; and that any discrepancies from the study as planned (and, if relevant, registered) have been explained.

Data sharing: No additional data available.

## Notes

Cite this as: *BMJ* 2015;351:h5774
